# Structural characterization and immunostimulant activities of polysaccharides fractionated by gradient ethanol precipitation method from *Panax ginseng* C. A. Meyer

**DOI:** 10.3389/fphar.2024.1388206

**Published:** 2024-04-24

**Authors:** Mengran Xu, Jing Ren, Ziye Jiang, Shuo Zhou, Enpeng Wang, Hui Li, Wei Wu, Xiaoyu Zhang, Jing Wang, Lili Jiao

**Affiliations:** ^1^ Jilin Ginseng Academy, Changchun University of Chinese Medicine, Changchun, China; ^2^ The Affiliated Hospital Changchun University of Chinese Medicine, Changchun University of Chinese Medicine, Changchun, China

**Keywords:** *Panax ginseng* C. A. Meyer, polysaccharide, immune, structure, gradient ethanol precipitation

## Abstract

*Panax ginseng* C. A. Meyer is a dual-purpose plant for medicine and food, its polysaccharide is considered as an immune enhancer. Four polysaccharides, WGP-20-F, WGP-40-F, WGP-60-F and WGP-80-F were obtained from ginseng via water extraction and gradient ethanol precipitation with different molecular weights (*Mw*) of 1.720 × 10^6^, 1.434 × 10^6^, 4.225 × 10^4^ and 1.520 × 10^4^ Da, respectively. WGP-20-F and WGP-40-F which with higher *Mw* and a triple-helix structure are glucans composed of 4-ɑ-Glc*p*, do not show remarkable immunoregulatory effects. WGP-60-F and WGP-80-F are heteropolysaccharides mainly composed of 4-ɑ-Glc*p* and also contain t-ɑ-Ara*f*, 5-ɑ-Ara*f* and 3,5-ɑ-Ara*f*. They are spherical branched conformations without a triple-helix structure and can effectively increase the index of immune organs, lymphocyte proliferation, activate macrophages to regulate the immune system in mice and further enhance immune functions by improving delayed-type hypersensitivity reaction and antibody response. These results indicated that WGP-60-F and WGP-80-F could be used as potential immune enhancers, and gradient ethanol precipitation can be applied for the preparation of ginseng bioactive polysaccharide.

## 1 Introduction

*Panax ginseng* C. A. Meyer is a medicinal and edible homologous plant and traditional Chinese medicine, widely used in cosmetics and food, especially in health foods (Jung et al., 2017; Lee et al., 2022). Its main active ingredient, ginseng polysaccharides, is a non-toxic side effect healthy food which is considered as an immune enhancer (Yang et al., 2022), can stimulate lymphocyte proliferation, promote the surface molecule expression of dendritic cells, induce dendritic cell maturation and increase the macrophage phagocytic capacity in terms of immune cell activity (Kim et al., 2009). In addition, the immunoregulatory activities of ginseng polysaccharides are closely related to molecular weight (*Mw*), sugar residue composition and polysaccharide domain (Yao et al., 2015a). Lemmon et al., 2012 found that the high *Mw* polysaccharides in North American ginseng are key immunomodulators. They likely trigger multiple signalling pathways in peripheral blood mononuclear cells, thereby inducing a T(h)1 transcriptional profile. Ginseng neutral polysaccharide (GPNE-I), mainly composed of glucan domain and type I and type II arabinogalactans (AG-I and AG-II), can effectively stimulate lymphocyte proliferation (Li et al., 2019).

Fractional precipitation is a classical separation method of polysaccharides, which is based on different solubilities of polysaccharides in different concentrations of ethanol or ketone solutions. The solubility of polysaccharides decreases with the increase in their *Mw* (Wang et al., 2019). The commonly used solvents for polysaccharide fractionation and precipitation are methanol, ethanol and acetone (Hu and Douglas, 2018). Ethanol is widely used because of its safety and food use. This method only requires simple equipment and has a wide range of applications for polysaccharide preparation. It can be used for mass preparation and industrial production (Hu and Douglas, 2018). At present, many methods such as equal concentration ethanol precipitation combined with ion exchange chromatography, gel chromatography and ultrafiltration membrane system, etc., have been used in research to obtain a variety of active polysaccharides with different structural characteristics from ginseng (Lee et al., 2019; Jiao et al., 2021; Zhang et al., 2021). However, the above methods for preparing active ginseng polysaccharides are relatively complex, simple and feasible preparation methods may be required in the large-scale preparation of active ginseng polysaccharides (Fernando et al., 2020). In contrast, the method of ethanol gradient precipitation can be used in preparing active ginseng polysaccharides and will simplify the preparation process of ginseng polysaccharides. Research shows that a variety of polysaccharides with different *Mw*, monosaccharide compositions, glycosidic bond types and chain conformations have been obtained by gradient ethanol precipitation (Wang et al., 2019; Liu Y. et al., 2022).

Therefore, this study intends to obtain ginseng polysaccharides with different fractions via gradient ethanol precipitation, and also aims to determine the physicochemical properties, primary structure, advanced conformation and *in vivo* immune activity of ginseng polysaccharides. Our results provide a simple and feasible technical strategy for preparing the ginseng active polysaccharides and enrich the useful data on the structure-activity relationship of ginseng polysaccharide and the development of ginseng polysaccharide health food.

## 2 Materials and methods

### 2.1 Experimental materials

Five-year-old ginseng was produced in Jian City, Jilin Province, China. It was identified by Professor Wang Shumin (College of Pharmacy, Changchun University of Chinese Medicine). The samples were stored in Jilin Ginseng Academy, Changchun University of Chinese Medicine.

Sepharose CL-6B was purchased from Cytiva Co., Ltd. (Washington, United States). Monosaccharide standard Galactose (Gal), Galacturonic acid (GalA), Glucose (Glc), Glucuronic acid (GlcA), Arabinose (Ara), Rhamnose (Rha), Mannose (Man), Xylose (Xyl), Fucose (Fuc) were purchased from Yuanye Biotechnology Co., Ltd. (Shanghai, China). lipopolysaccharide (LPS), Concanavalin A (ConA) were obtained from Sigma Chemical Co. (St. Louis, United States). Methylthiazolyldiphenyl-tetrazolium bromide (MTT) was purchased from Beyotime Biotechnology (Shanghai, China). Nitric Oxide (NO) test kits were purchased from Nanjing Jiancheng Bioengineering Institute (Nanjing, China).

### 2.2 Experimental method

#### 2.2.1 Preparation of ginseng polysaccharides

The ginseng (500 g) was extracted thrice with 5 L of distilled water for three times when slightly boiled for 2 h each time. The extracts were combined, concentrated and precipitated by adding 20%, 40%, 60%, and 80% ethanol for ethanol precipitation at 4°C for 24 h. The samples were named WGP-20, WGP-40, WGP-60 and WGP-80. WGP-20, WGP-40, WGP-60, and WGP-80 were fractionated via gel permeation chromatography on Sepharose CL-6B column (3.0 cm × 100 cm) at a flow rate of 0.5 mL/min. The mobile phase was physiological saline, and the purified fractions WGP-20-F, WGP-40-F, WGP-60-F and WGP-80-F were obtained. The total carbohydrate content of WGPs was determined via the phenol–sulphuric acid method (DuBois et al., 1956). The uronic acid of WGPs was determined by the m-hydroxydiphenyl method (Blumenkrantz and Asboe-Hansen, 1973). The protein contents were determined by ultraviolet (UV) spectrophotometer within the 190–400 nm range.

#### 2.2.2 Homogeneity analysis

The homogeneity of WGPs was determined via high performance gel permeation Chromatography (HPGPC) on a Diane UniMate 3000 system (Diane, United States) with TSK-G3000 PWXL columns (7.8 mm × 30.0 cm) (Li et al., 2019). The 1 mg/mL sample was filtered and tested, with a flow rate of 0.6 mL/min, a temperature of 40°C, and a mobile phase of ultrapure water.

#### 2.2.3 Monosaccharide compositions analysis

Firstly, acid hydrolysis was carried out, and 4 mg of polysaccharide was added to the sample bottle. 2 mol/L of hydrochloric acid methanol was added and hydrolysed at 80°C for 16 h. Anhydrous ethanol was used to remove the excessive hydrochloric acid. The hydrolysate was mixed with 0.3 mol/L of NaOH and 0.5 mol/L of PMP methanol solution, the mixture was subjected to a water bath at 70°C for 30 min, and cooled to 25°C. The resulting solution was neutralized with 0.3 mol/L of HCl. Subsequently, the PMP derivative was obtained by extraction with equal volume of chloroform for 4 times, filtered with 0.22 μm the membrane, and analysized using the Dionex UniMate3000 HPLC system (Thermo Fisher Scientific, United States) with an Inertsil ODS-3 column (4.6 ID × 250 mm). The eluent was phosphate buffer (0.1 mol/L; pH 7.0) and acetonitrile (83:17, v/v), with a flow rate of 1.0 mL/min, a column temperature of 40°C and the injection volume was 10 μL (Zhang et al., 2009).

#### 2.2.4 Structural analysis of ginseng polysaccharides

##### 2.2.4.1 Fourier-transform infrared spectroscopy (FT-IR)

WGPs (2 mg) were mixed with KBr to prepare a KBr pellet pressing and recorded on a Bruker (Germany) Vertex 7.0 FT-IR spectrometer within the 4000–400 cm^−1^ range, 32 scans per scan.

##### 2.2.4.2 Methylation and GC-MS analysis

WGPs were methylated according to the method of Gong et al. (2017). Approximately 10 mg of samples was dissolved in 10 mL DMSO. The DMSO/NaOH suspension which was prepared from 1 mL DMSO and 100 μL 0.05 mg/mL NaOH, was added and stirred for 1 h. Then, 3.6 mL of methyl iodide was added to obtain an accurate reaction for 7 min. The reaction was stopped by adding 6 mL of distilled water. The product was dialysed and dried under reduced pressure at 40°C. Methylated polysaccharides were extracted by chloroform. Then, 6 μg inositol and 1 mL trifluoroacetic acid were added into fully methylated polysaccharide at 120°C for 2 h. It was dried with nitrogen and reduced by 70 mg NaBH_4_ at 25°C. Then, 0.5 mL acetic anhydride and 0.5 mL anhydrous pyridine were added and acetylated at 100°C for 2 h. The obtained sample was dissolved with a little chloroform and analysed via GC-MS (TSQ 8000, Thermo Fisher Scientific, United States) with a TG-5SiLMS column (30 m × 0.25 mm).

##### 2.2.4.3 NMR spectroscopy analysis

WGPs (30 mg) were dissolved in D_2_O (0.5 mL, 99.9%). ^1^H NMR and ^13^C NMR spectra were recorded at 25°C on Bruker AV600 MHz NMR spectrometer (Germany).

##### 2.2.4.4 Congo red experiment

First, 2 mL of 2 mg/mL polysaccharide solution was blended with the same amount of 91 μmol/L Congo red reagent. Then, 1 mL NaOH (0–0.7 mol/L) of different concentrations was added to the mixture and the Congo red solution without polysaccharide. The absorbance value was recorded at 400–700 nm using a UV spectrophotometer.

##### 2.2.4.5 CD spectroscopy assay

The polysaccharides were dissolved in distilled water (1 mg/mL). The circular dichroic spectrophotometer (Chirascan Qcd, Applied Photophysics Co., Ltd., Leatherhead, Surry, United Kingdom) was used at 25°C with a bandwidth of 1.0 nm. The data were recorded within the 190–350 nm range at a scanning speed of 20 nm/min.

##### 2.2.4.6 Size-exclusion chromatography–multiangle laser light scattering

The *Mw* and configuration of polysaccharides were analysed using the SEC-MALLS system. The chromatographic column (OHpak SB-803HQ column, 8.0 × 300 mm, Shodex, Japan) was eluted with 0.2 mol/L NaCl solution containing 0.02% NaN_3_ at a flow rate of 0.6 mL/min at 35°C. The refractive index increment (DN/DC) was 0.185 mL/g. The polysaccharide solution (0.5 mg/mL) was treated with a 0.22 μm filter membrane. The number average molecular weight (*Mn*), weight average molecular weight (*Mw*), partition coefficient (*Mw*/*Mn*) and conformation of each fraction were analysed using Astra software.

##### 2.2.4.7 Scanning electron microscopy analysis

The micrographs of WGPs were sputtered with gold under reduced pressure and obtained via field emission SEM (Quanta 200, America) at a 3 kV acceleration voltage.

#### 2.2.5 Immune activity of ginseng polysaccharides

##### 2.2.5.1 Animal treatment

Healthy 4-week-old C57BL/6 male mice (15 ± 2 g) were cultured at 25°C of 20°C–24°C, relative humidity of 50%–60% and light conditions (12:12 h: light and dark cycle). The mice were freely fed with a standard diet and water. All animals were maintained and used in strict accordance with the PR China research Council’s Guide for the Care and Use of Laboratory Animals and with the guidelines issued by the Experimental Animal Centre of Changchun University of Traditional Chinese Medicine. This study was approved by the university committee for animal experiments. The number and date of approval of the program by the Ethics Committee for this research was 2021030 and March 2021.

The animal experiment was divided into three parts. Each part of the mice was divided into 14 groups, with 10 mice in each group. The mice in the polysaccharide treatment group were given 0.2 mL of WGPs with different concentrations (50, 100, and 200 mg/kg) by oral gavage. The mice in negative control groups were administrated 0.2 mL of normal saline orally. All mice were continuously given WGPs or normal saline for 30 days.

##### 2.2.5.2 Effects of ginseng polysaccharide on immune organs and body weight of mice

On the next day after the last administration, all of the mice were sacrificed by cervical dislocation, and their eyeballs were removed to collect blood samples. The required spleen and thymus were taken out under sterile conditions. They were quickly placed in the glass petri dish. Then, the residual blood on the tissue surface was washed with D-Hanks’ solution. The immune index was weighed and calculated.

##### 2.2.5.3 Effect of ginseng polysaccharide on splenic lymphocyte proliferation in mice

The mice’s spleen was taken out under aseptic conditions. The spleen lymphocytes were extracted according to the previous report (Wang et al., 2018). The number of cells and cell survival rate were determined using the trypan blue staining method. The cells were diluted to 1 × 10^6^ pieces/mL to prepare a single-cell suspension of splenic lymphocytes.

Approximately 100 μL of the prepared splenic lymphocyte suspension was added to each cell culture plate well. ConA (5 μg/mL) or LPS (20 μg/mL) were added. The blank control and experimental groups were set up and cultured at 37°C in a 5% CO_2_ incubator for 68 h. Then, 20 μL MTT solution was added into the wells and incubated at 37°C for 4 h. Afterwards, 100 μL supernatant was discarded, and 150 μL of DMSO was added into the wells. The absorbance at 570 nm was determined.

##### 2.2.5.4 Effect of ginseng polysaccharides on spleen NK cells

Mice spleen lymphocytes were treated as effector cells. YAC-1 cells were treated as target cells. The cells were cultured in 96 well plates, which were divided into three regions and into experimental groups (each well was added with 100 μL YAC-1 cells and 100 μL splenocyte suspension), blank control group (each well was added with 100 μL YAC-1 cells and 100 μL complete medium) and maximum release group (each well was added with 100 μL YAC-1 cells and 100 μL 1% NP40). They were cultured at 37°C in a 5% CO_2_ incubator for 44 h. After centrifugation, 100 μL supernatant was removed from each well and placed in the culture plate. Then, 100 μL LDH matrix solution was added simultaneously to obtain the reaction for 8 min. Afterwards, 30 μL of 1 mol/L HCl was added to each well. The absorbance at 490 nm was determined (He et al., 2017).

##### 2.2.5.5 Effect of ginseng polysaccharide on phagocytosis of peritoneal macrophages

The mice were treated for 30 days according to the feeding method discussed in Section 2.2.5.1. The macrophages were extracted from the mice’s abdominal cavities. The experiments were conducted similarly to the reported methods (He et al., 2017). Macrophages were examined using the trypan blue staining method to measure a 95% survival rate for assay.

After the purification of macrophages, culturing was continued for 24 h, and the supernatant was removed. Then, 100 μL neutral red solution (0.075%) was added to each well. Culturing was continued for 1 h. The neutral red was washed with PBS. Then, 100 μL cell lysate (ethanol: acetic acid = 1:1 v/v) was added to each well for 24 h. The absorbance at 550 nm was determined.

##### 2.2.5.6 Effect of ginseng polysaccharides on the activity of NO

The macrophages were separated according to Section 2.2.5.5. Culturing was continued for 24 h. After centrifugation, the supernatant was removed. The contents of NO were measured according to the instructions from the kit. The absorbance at 550 nm was determined.

##### 2.2.5.7 Effect of ginseng polysaccharides on delayed-type hypersensitivity in mice

The mice were treated for 30 days according to the feeding method discussed in Section 2.2.5.1. The sheep blood was washed with normal saline thrice and centrifuged (2000 r/min) for 10 min each time. The defibrinated sheep red blood cell (SRBC) was prepared into 2% (v/v) cell suspension with normal saline. On the 2nd day after the last administration of the polysaccharides, 0.2 mL was injected intraperitoneally into each mouse (except the control group). After 4 days, the thickness of the left voix pedis was measured. Then, 20 µL of 20% (v/v) SRBC was injected subcutaneously at the measuring site. After 24 h, the thickness of the left rear sole was measured again. The degree of DTH was expressed by the difference in the voix pedis thickness (voix pedis swelling) before and after the attack.

##### 2.2.5.8 Determination of plaque-forming cell (PFC) assay in mouse spleen

After measuring DTH, the mice were killed. Then, the spleen was taken aseptically. The spleen cells were separated using Hanks’ solution. The number of cells and cell survival rate were determined using the trypan blue staining method. Then, the cell concentration was adjusted to 5 × 10^6^ pieces/mL. The surface culture medium was heated, dissolved and mixed with the same amount of Hanks’ solution of twice the concentration in PH 7.2. Each tube was divided into approximately 0.5 mL and 50 µL of 10% SRBC with salicylic acid (SA) solution. Then, 20 µL splenocyte suspension was added into the tubes. After being thoroughly mixed, it was poured onto the thin layer of agarose slide. After the agarose was solidified, the slide was buckled flat on the slide frame and placed into the CO_2_ incubator for 1.5 h. The complement diluted with SA buffer solution (1:8) was added into the slide groove and continued to be incubated for 1.5 h. Then, the number of plaques was expressed as the number of PFC per 10^6^ viable splenocytes.

## 3 Results

### 3.1 The primary structure of WGPs

#### 3.1.1 Characteristics of WGPs

**FIGURE 1:**
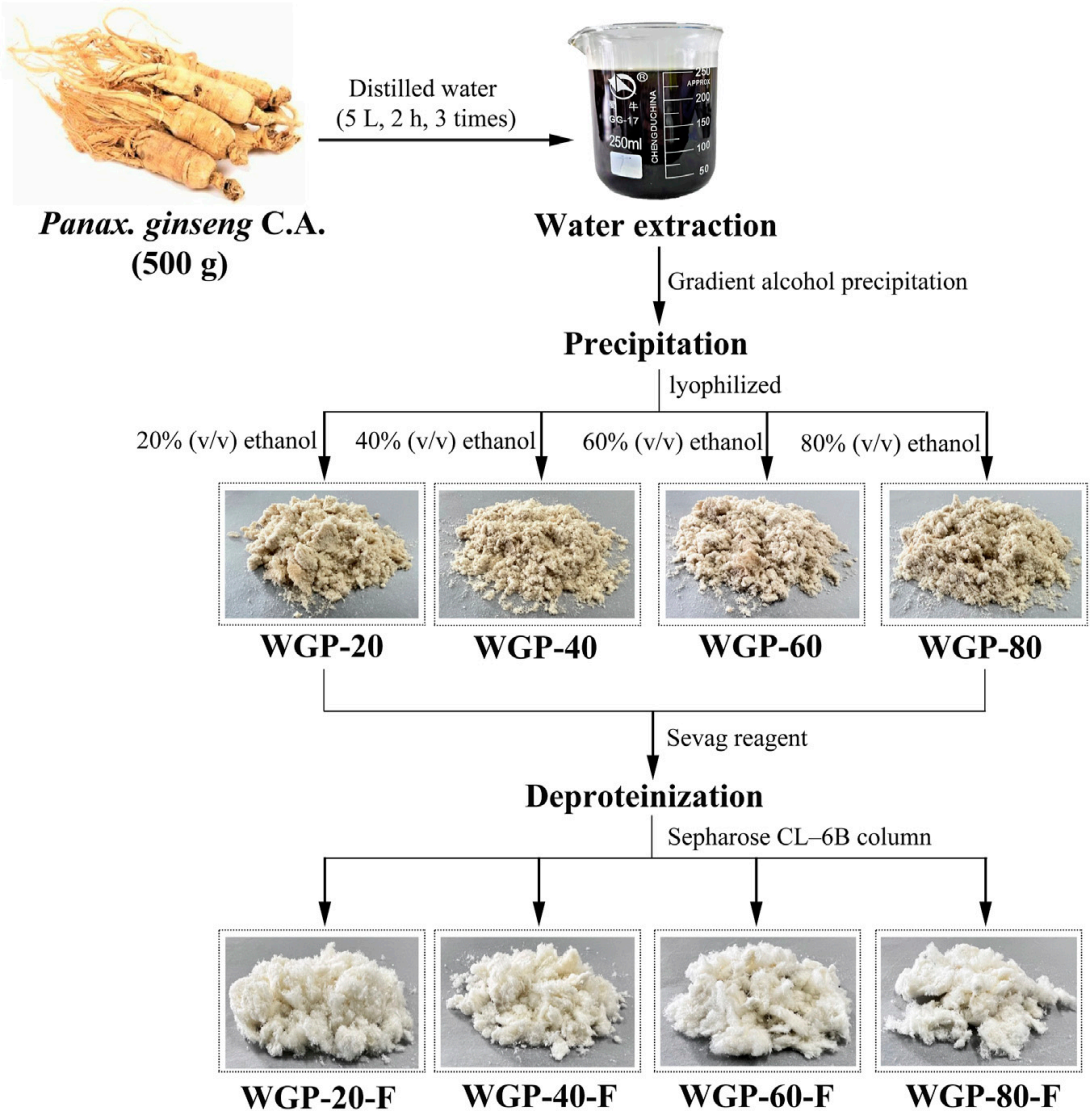
Fractionation procedure of the polysaccharides.

**FIGURE 2:**
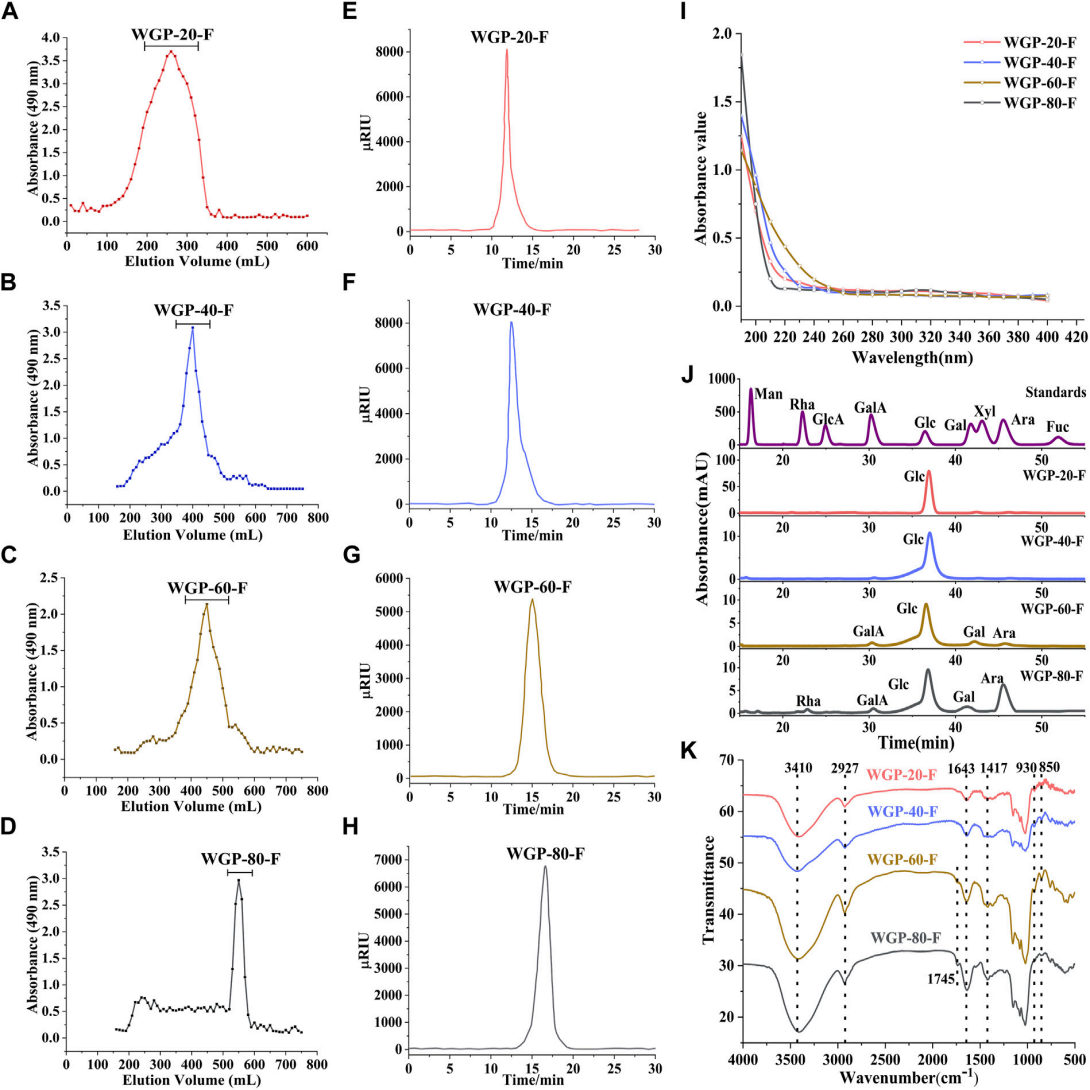
Characterisation of WGPs. **(A–D)**: Sepharose CL-6B gel permeation chromatography profile of WGPs; **(E–H)**: HPLC chromatography of WGPs; **(I)**: UV spectra of WGPs; **(J)**: Monosaccharide analysis of WGPs; **(K)**: FT-IR spectra of WGPs.

After hot water extraction, gradient ethanol precipitation and purification using Sepharose CL-6B column were applied, the four fractions, WGP-20-F, WGP-40-F, WGP-60-F and WGP-80-F, were obtained from crude ginseng polysaccharides (Figure 1). The yields, carbohydrate content, uronic acid content, *Mw* and Monosaccharide were showed in Table 1. It can be seen that the Carbohydrate content of the four polysaccharides were more than 90%, with high purity. WGP-60-F and WGP-80-F contain a certain amount of uronic acid. At the same time, with the increase of ethanol concentration, the *Mw* of WGPs showed a descending order: WGP-20-F > WGP-40-F > WGP-60-F > WGP-80-F. WGPs showed a single and symmetrical elution curve in HPGPC. This finding indicated they were homogeneous (Figures 2A–H).

**TABLE 1:** Chemical composition of WGPs.

Item	WGP-20-F	WGP-40-F	WGP-60-F	WGP-80-F
Yield (%)^A^	40.46 ± 3.28^a^	11.09 ± 1.33^b^	2.84 ± 0.28^d^	5.41 ± 0.43^c^
Yield (%)^B^	70.12 ± 4.43^a^	63.14 ± 5.79^b^	60.98 ± 3.58^b^	62.83 ± 4.22^b^
Carbohydrate (%)	92.32 ± 1.46^a^	94.25 ± 1.05^a^	94.27 ± 2.15^a^	93.58 ± 2.47^a^
Uronic acid (%)	0.27 ± 0.20^c^	0.19 ± 0.23^d^	3.45 ± 0.16^a^	2.16 ± 0.18^b^
Protein (%)	0.67 ± 0.23^a^	0.45 ± 0.17^b^	0.73 ± 0.11^a^	0.64 ± 0.31^a^
Molecular weight (Da)	1.720 × 10^6^ ^a^	1.434 × 10^6^ ^b^	4.225 × 10^4^ ^c^	1.520 × 10^4^ ^d^
Monosaccharide composition (molar ratio, %)				
Glc	100	100	88.83	52.09
Gal	—	—	5.33	2.89
GalA	—	—	3.50	2.31
Ara	—	—	2.34	40.66
Rha	—	—	—	2.05

^A^ : The yield of WGPs, were relative to dry weight of ginseng.

^B^ : The yield of WGP-20-F, WGP-40-F, WGP-60-F and WGP-80-F was derived from WGP-20, WGP-40, WGP-60, and WGP-80.

“—“: undetected. Not: Data were presented as M ± SD (*n* = 3). Means marked with different superscript alphabets in the same row indicate significant differences (*p* < 0.05).

No evident absorption peaks were found at 280 nm in the UV spectra of four polysaccharides, as shown in Figure 2I.

The monosaccharide composition analysis exhibited that WGP-20-F and WGP-40-F were composed of Glc only. WGP-60-F and WGP-80-F were acid heteropolysaccharides. with a GalA content of 3.50% and 2.31%, respectively. With its content reaching 88.83%, Glc was still the main component in WGP-60-F. It also consisted of a small amount of Gal (5.33%), Ara (2.34%) and GalA (3.50%). WGP-80-F was composed of Glc, Ara, Gal and Rha with 52.09%, 40.66%, 2.89% and 2.05% (Figure 2J).

#### 3.1.2 FT-IR analysis of WGPs

The FT-IR spectra of WGPs are shown in Figure 2K. The strong absorption peak near 3410 cm^−1^ represents the O-H vibration peaks. The absorption peaks near 2927 and 1643 cm^−1^ are caused by the C-H bending vibration peaks and O-H stretching vibration peaks, respectively. The absorption peak in the 1000–1200 cm^−1^ range represents the existence of the pyran sugar ring. The characteristic absorption peak at 930 cm^−1^ belongs to the β-glycoside bond. Moreover, the characteristic absorption peak at 850 cm^−1^ belongs to the α-glycoside bond. The results show that WGPs exist simultaneously in the α-glycoside bond and β-glycoside bond connection mode (Wang et al., 2018).

#### 3.1.3 Methylation analysis of WGPs

Methylation and GC-MS analysis were used to investigate the glycosidic linkage of WGPs. As listed in Table 2, the glycosidic bond and the molar ratio of the sugar residues were analysed by comparing the ion mass spectra of partially methylated alditol acetates, their retention times and the peak area in the GC chromatogram. The dominant sugar residue in the four polysaccharides was 4-Glc*p*. For WGP-20-F, four types of linkages were identified: t-Glc*p*, 4-Glc*p*, 3,4-Glc*p* and 3,6-Glc*p* with a molar ratio of 6.61:5.88:1.48:1. WGP-40-F contained five sugar residues: t-Glc*p*, 3-Glc*p*, 4-Glc*p*, 3,4-Glc*p* and 4,6-Glc*p*, with a molar ratio of 2.47:1:2.45:1.37:1.04. Unlike WGP-20-F and WGP-40-F, WGP-60-F mainly contained six types of linkages, whereas WGP-80-F comprised nine types. WGP-60-F and WGP-80-F contained t-Ara*f*, t-Glc*p*, 5-Ara*f*, 4-Glc*p*, 3,4-Glc*p* and 4,6-Gal*p* molar ratios of 1.06:3.24:1:24.14:1.75:3.41 and 4.12:1.58:3:82:11.5:1.35:1, respectively. Moreover, WGP-80-F also contained 3,5-Ara*f*, 2,4-Rha*p* and 3,6-Glc*p*. The results of methylation were consistent with those of monosaccharide composition.

**TABLE 2:** Methylation analysis of WGPs.

Methylated sugars	Linkages type	Relative amount (mol%)			
		WGP-20-F	WGP-40-F	WGP-60-F	WGP-80-F
2,3,5-Me_3_-Ara^*^	Ara*f*-(1→	—	—	3.07	13.46
2,3-Me_2_-Ara	→5)-Ara*f*-(1→	—	—	2.89	12.50
2,3,4,6-Me_4_-Glc	Glc*p*-(1→	44.15	29.69	9.37	5.18
2-Me_1_-Ara	→3,5)-Ara*f*-(1→	—	—	—	15.11
2,4,6-Me_3_-Glc	→3)-Glc*p*-(1→	—	12.04	—	—
3-Me_1_-Rha	→2,4)-Rha*p*-(1→	—	—	—	2.65
2,3,6-Me_3_-Glc	→4)-Glc*p*-(1→	39.31	29.49	69.76	37.61
2,6-Me_2_-Glc	→3,4)-Glc*p*-(1→	9.86	16.47	5.05	4.40
2,3- Me_2_-Glc	→4,6)-Glc*p*-(1→	—	12.51	—	—
2,3- Me_2_-Gal	→4,6)-Gal*p*-(1→	—	—	9.86	3.27
2,4- Me_2_-Glc	→3,6)-Glc*p*-(1→	6.68	—	—	5.82

^*^2,3,5-Ara: 1,4-di-O-acetyl-2, 3,5-tri-O-methyl-Arabitol.

“—“: undetected.

#### 3.1.4 NMR spectroscopy of WGPs

**FIGURE 3:**
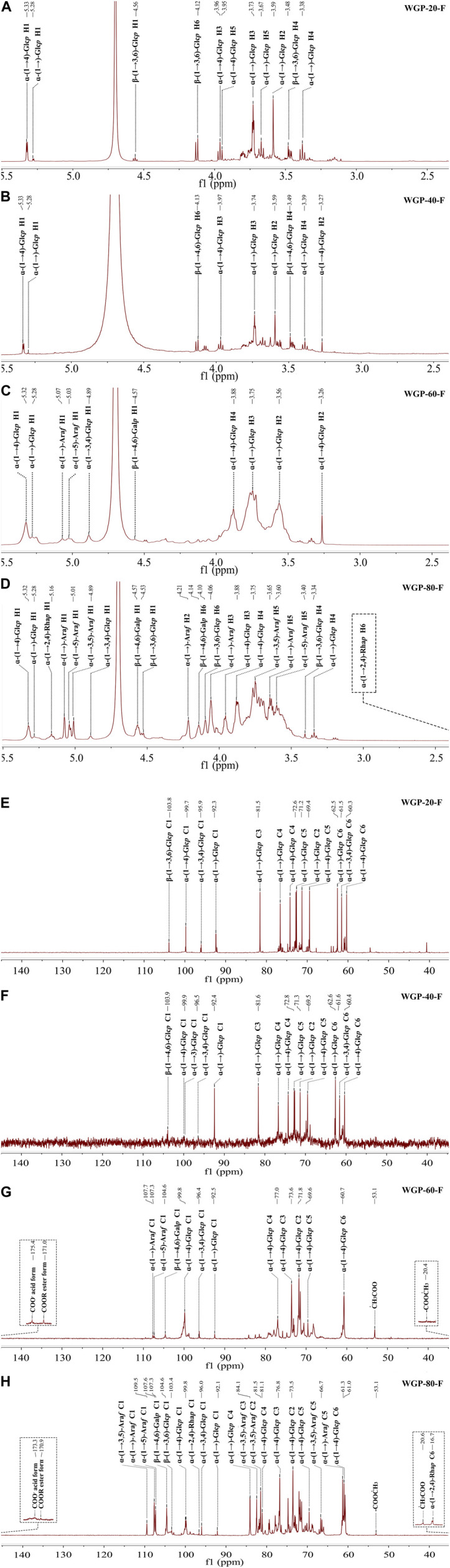
(Continued).

In the ^1^H NMR WGPs, the spectra of all the subfractions were similar, although the peak intensity was different. The anomeric region of WGPs (Figures 3A–D) appeared within the 4.5–4.6 ppm (β-) and 4.8–5.4 ppm (α-) ranges (Agrawal, 1992; Liao et al., 2015). The strong signals around 5.3, 5.28, 4.89, 4.57, and 4.56 ppm were assigned to the anomeric proton of (1→4)-α-Glc*p*, t-α-Glc*p*, (1→3,4)-α-Glc*p*, (1→4,6)-β-Glc*p* and (1→3,6)-β-Glc*p*. In the spectra of WGP-60-F and WGP-80-F (Figures 3G,H), the strong signals at 5.07 and 5.03 ppm were assigned to the anomeric proton of t-α-Ara*f* and (1→5)-α-Ara*f*. In the spectra of WGP-80-F (Figure 3H), the strong signal at 5.01 ppm was attributed to the H-1 of (1→3,5)-α-Ara*f*. The strong signals observed at 5.16 and 1.17 ppm were assigned to the anomeric proton and protons of H-6 derived from (1→2,4)-α-Rha*p*, respectively (Agrawal, 1992), which were combined with the methylation results.

The anomeric carbon signals of WGPs (Figures 3E–H) appeared within the 92.1–109.5 ppm range. This finding also indicated that the polysaccharides contained α-configuration and β-configuration. In the ^13^C NMR spectra, the strong signal around 92.1–92.5, 95.9–96.5, 99.7–100.0, 103.3–104.0, 104.5–104.6, 107.3–107.4, 107.6–107.7, and 109.5 ppm were assigned to the anomeric carbon of t-α-Glc*p*, (1→3,4)-α-Glc*p*, (1→4)-α-Glc*p*, (1→3,6)-β-Glc*p*, (1→4,6)-β-Glc*p*, (1→5)-α-Ara*f*, t-α-Ara*f* and (1→3,5)-α-Ara*f*, respectively. In the ^13^C NMR spectra of WGP-60-F and WGP-80-F (Figures 3C,D), the signals around 175.3–175.4, 170.9–171.0, 53.1–53.2, and 20.4–20.7 ppm were caused by the carboxyl carbons of the unesterified, methyl-esterified carbonyls, methyl and acetyl groups attached to α-GalA*p*. The strong signals at 96.6 and 16.7 ppm were assigned to anomeric carbons and C-6 of (1→2,4)-α-Rha*p*, respectively, in the ^13^C NMR spectra of WGP-80-F (Agrawal, 1992).

The complex, overlapping signals at 16.7–85.0 ppm and 1.17–4.21 ppm were attributed to C-2 to C-6 groups and H-2 to H-6 groups of different linkages in WGPs.

### 3.2 Advanced conformation of WGPs

#### 3.2.1 Congo red analysis of WGPs

**FIGURE 4:**
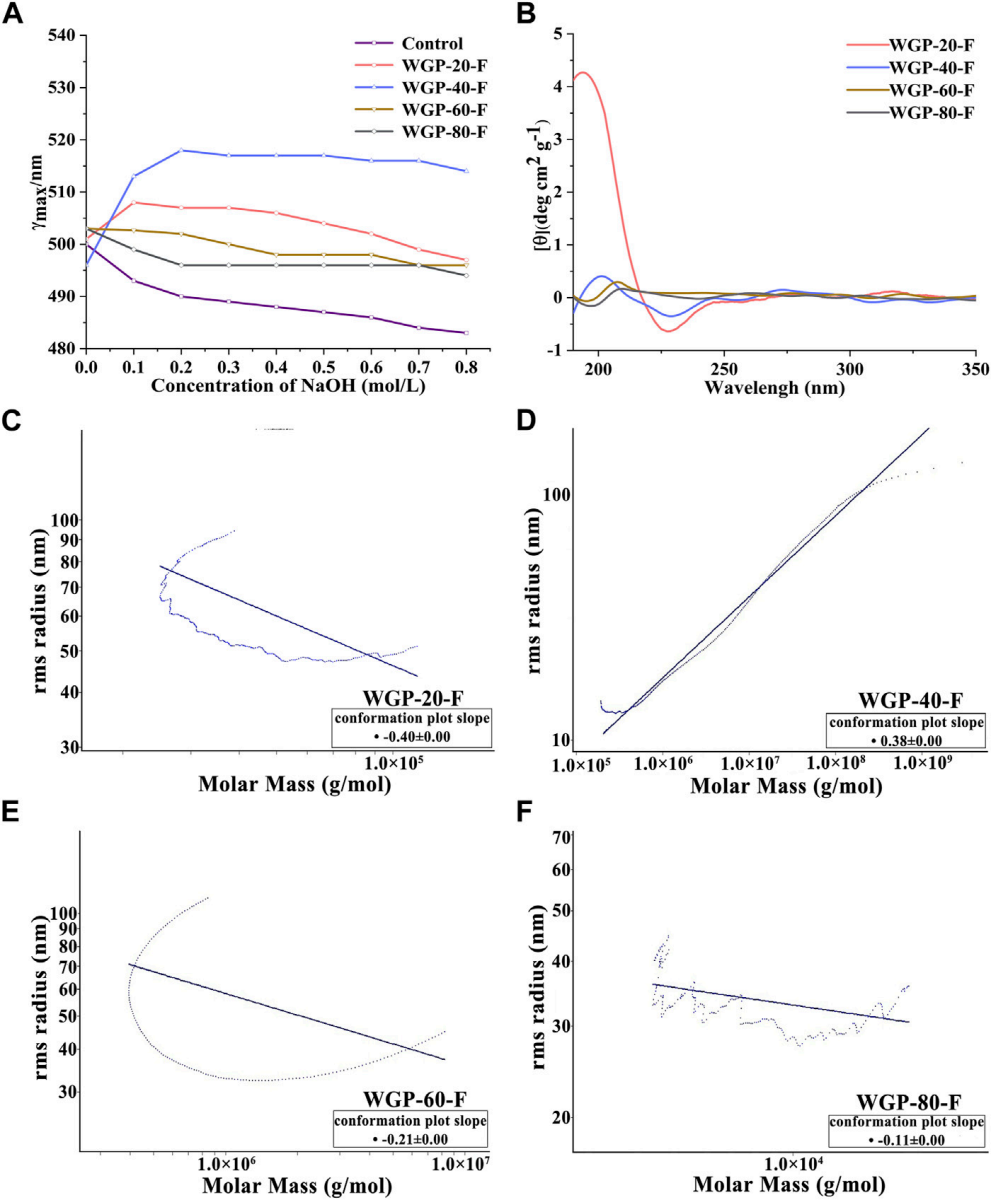
Higher conformation of WGPs. **(A)** Changes in absorption maximum of Congo red–WGPs complex at various concentrations of NaOH; **(B)** CD spectra of WGPs; **(C–F)**: SEC-MALLS chromatograms by MALLS of WGPs.

Congo red is a polymer dye that can form a complex with polysaccharides with triple-helix chain conformation. The changes in the maximum absorption wavelength of WGPs with Congo red in 0–0.8 mol/L NaOH solution are shown in Figure 4A. The redshifts of the λmax were observed at 508 and 518 nm for WGP-20-F and WGP-40-F, respectively. The redshifts were higher than those for the Congo red solution, suggesting the presence of triple-helix structure in WGP-20-F and WGP-40-F (Liu G. et al., 2022). On the contrary, the WGP-60-F and WGP-80-F Congo red solution did not show a bathochromic shift with respect to Congo red solution. This finding indicated that WGP-60-F and WGP-80-F have no triple-helix structure, and these four polysaccharides possessed different conformations.

#### 3.2.2 CD spectroscopy analysis of WGPs

The CD is an optical method for studying the asymmetry of the three-dimensional molecular spatial structure of biological macromolecules (Bystrický et al., 1995). As shown in Figure 4B, the CD spectra of WGP-20-F and WGP-40-F showed positive and negative Cotton effects at around 198 and 228 nm, respectively. This finding indicated the influence of a helix-like structure (Tatiane et al., 2010). Compared with WGP-20-F and WGP-40-F, the positive and negative Cotton effects of WGP-60-F and WGP-80-F were detected around 208 and 196 nm, thereby proving that the ordered spiral structure was weakened. The above results were consistent with Congo red staining results. Furthermore, they proved that the four polysaccharides had different chain conformations.

#### 3.2.3 SEC-MALLS analysis of WGPs

The SEC-MALLS system was used to analyse polysaccharides further. As shown in Figures 4C–F and Table 3, the *Mw* values of WGP-20-F, WGP-40-F, WGP-60-F and WGP-80-F were 1.720 × 10^6^, 1.434 × 10^6^, 4.225 × 10^4^, and 1.520 × 10^4^ Da, respectively. The distribution coefficients *Mw/Mn* of WGPs were close to 1, belonging to the sample with narrow distribution. The slope was the linear fitting slope of the Rg-*Mw* diagram of macromolecule conformation. According to the literature, it is a linear and irregular coil when the slope of the polysaccharide molecule is 0.4–0.6. When the slope is 0.33, it is spherical. An extension structure exists when the slope is greater than 0.6. However, the structure is a rod arrangement when the slope is 1. The results of this study are shown in Table 3. The slopes of WGP-20-F, WGP-40-F, WGP-60-F and WGP-80-F were 0.4, 0.38, 0.21 and 0.12, respectively. Thus, WGP-20-F and WGP-40-F were flexible linear polymers, whereas WGP-60-F and WGP-80-F were spherical with a high-branched structure (Li et al., 2014; Yan et al., 2019). In addition, the <S^2^>_Z_^1/2^–*Mw* relationship curves of WGP-60-F and WGP-80-F were U shaped (Figures 4E,F). This finding further confirmed that they were highly branched polysaccharides. This U-shaped distribution is a typical feature of highly branched polymers (Zhao et al., 2014; Zeng et al., 2016).

**TABLE 3:** SEC-MALLS analysis of WGPs.

Group	*Mw* (Da)	Mn	Mw/Mn	Mz	Slope
WGP-20-F	1.720×10^6^	1.444×10^6^	1.091	1.807×10^7^	0.40
WGP-40-F	1.434×10^6^	7.420×10^5^	1.132	3.177×10^6^	0.38
WGP-60-F	4.225×10^4^	3.613×10^4^	1.169	5.186×10^4^	0.21
WGP-80-F	1.520×10^4^	1.274×10^4^	1.193	2.021×10^4^	0.12

#### 3.2.4 SEM analysis of WGPs

**FIGURE 5:**
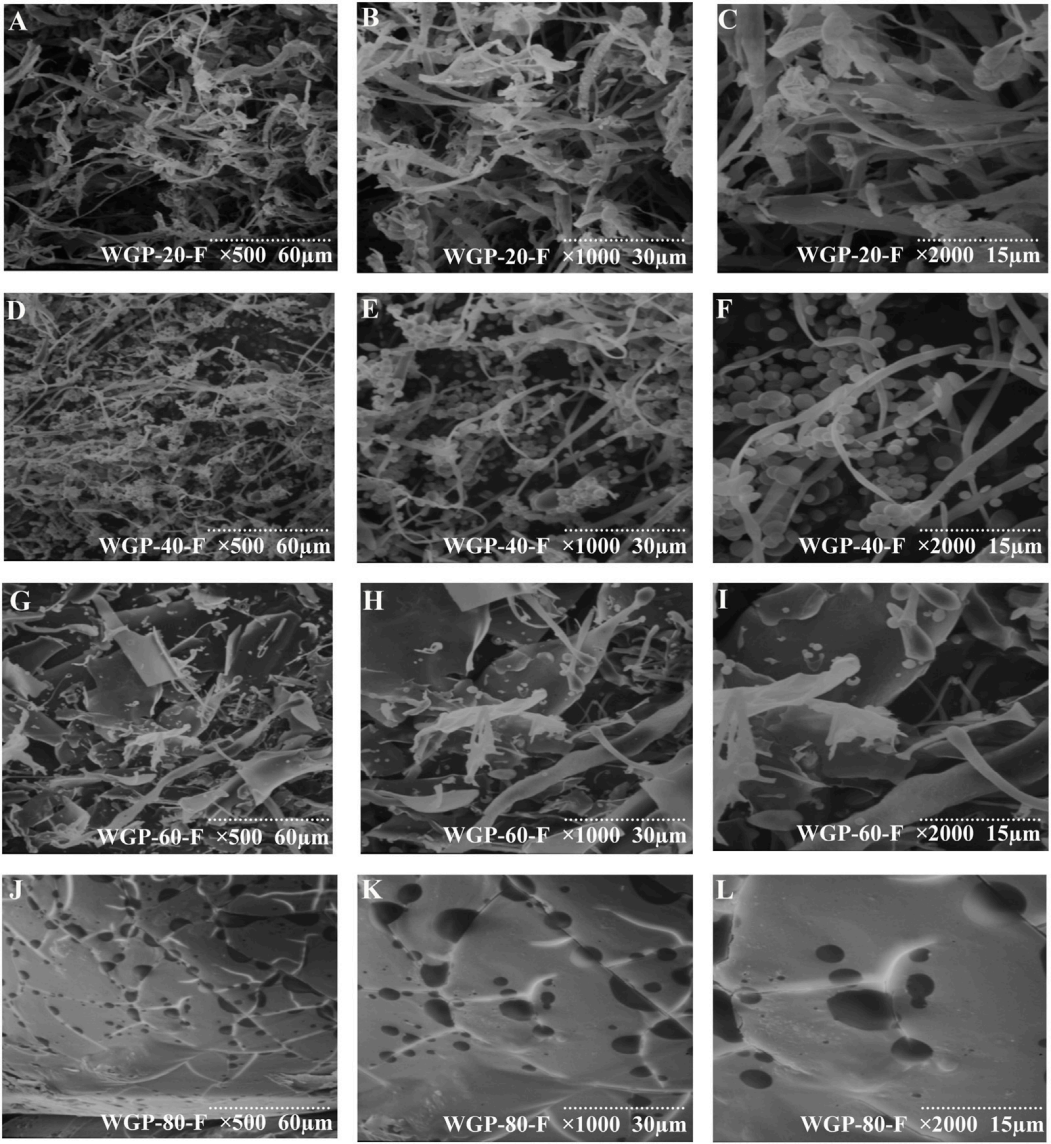
SEM analysis. **(A–C)** SEM of WGP-20-F at ×500, ×1000, ×2000 magnification; **(D–F)** SEM of WGP-40-F at ×500, ×1000, ×2000 magnification; **(G–I)** SEM of WGP-60-F at ×500, ×1000, ×2000 magnification; **(J–L)** SEM of WGP-80-F at ×500, ×1000, ×2000 magnification.

The scanning electron micrograph of WGPs is shown in Figure 4. WGP-20-F presented an irregular fibrous strip (Figures 5A–C) (Su and Li, 2020). WGP-40-F exhibited an irregular fibrous strip with a small globular structure (Figures 5D–F). WGP-60-F and WGP-80-F were different from WGP-20-F and WGP-40-F. In particular, the surface of WGP-60-F was shown as a sheet structure, and WGP-80-F showed a smooth surface but with distinct pore openings (Figures 5G–L) (Hu et al., 2020).

### 3.3 Immunological activity of WGPs

#### 3.3.1 Effect of WGPs on the body weight and immune index of mice

**FIGURE 6:**
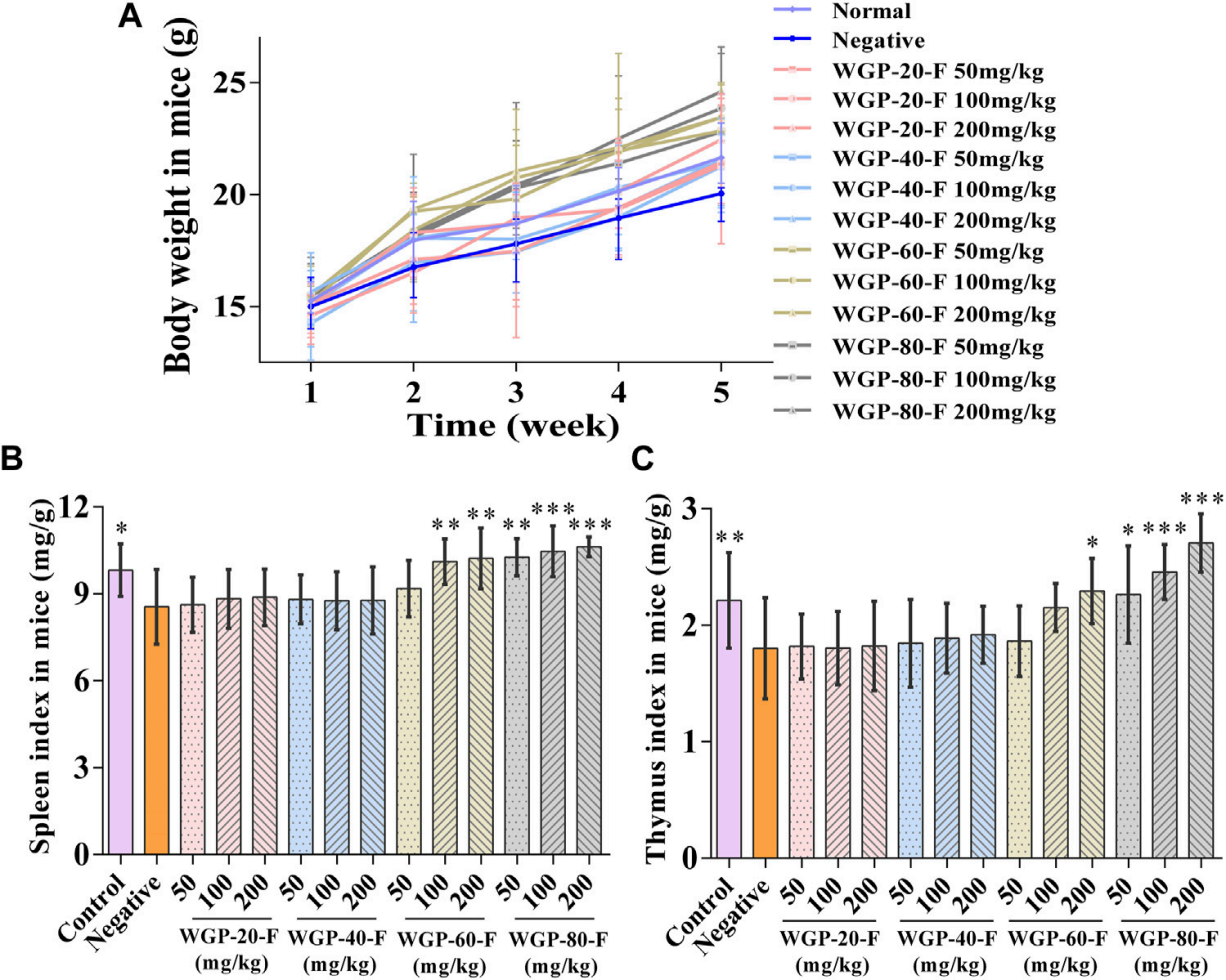
Effect of WGPs on body weight and immune index of mice. **(A)** Body weight; **(B)** Spleen index; **(C)** Thymus index. Every column represents the Mean ± SD. ***p < 0.001, **p < 0.01, *p < 0.05 *versus* negative group.

As shown in Figures 6A–C, compared with those in the negative control group, the body weight, spleen index and thymus index in mice in each group treated with WGP-60-F and WGP-80-F showed a significantly increasing trend in a dose-dependent manner (*p* < 0.01), particularly the administration group of WGP-80-F in 200 mg/kg shows the best effect, the spleen index and thymus index increased by 24.25% and 50.13%, respectively (*p* < 0.001). However, WGP-20-F and WGP-40-F groups exhibited no significant difference in the effect (*p* > 0.05).

#### 3.3.2 Effect of WGPs on the proliferation of spleen lymphocytes

**FIGURE 7:**
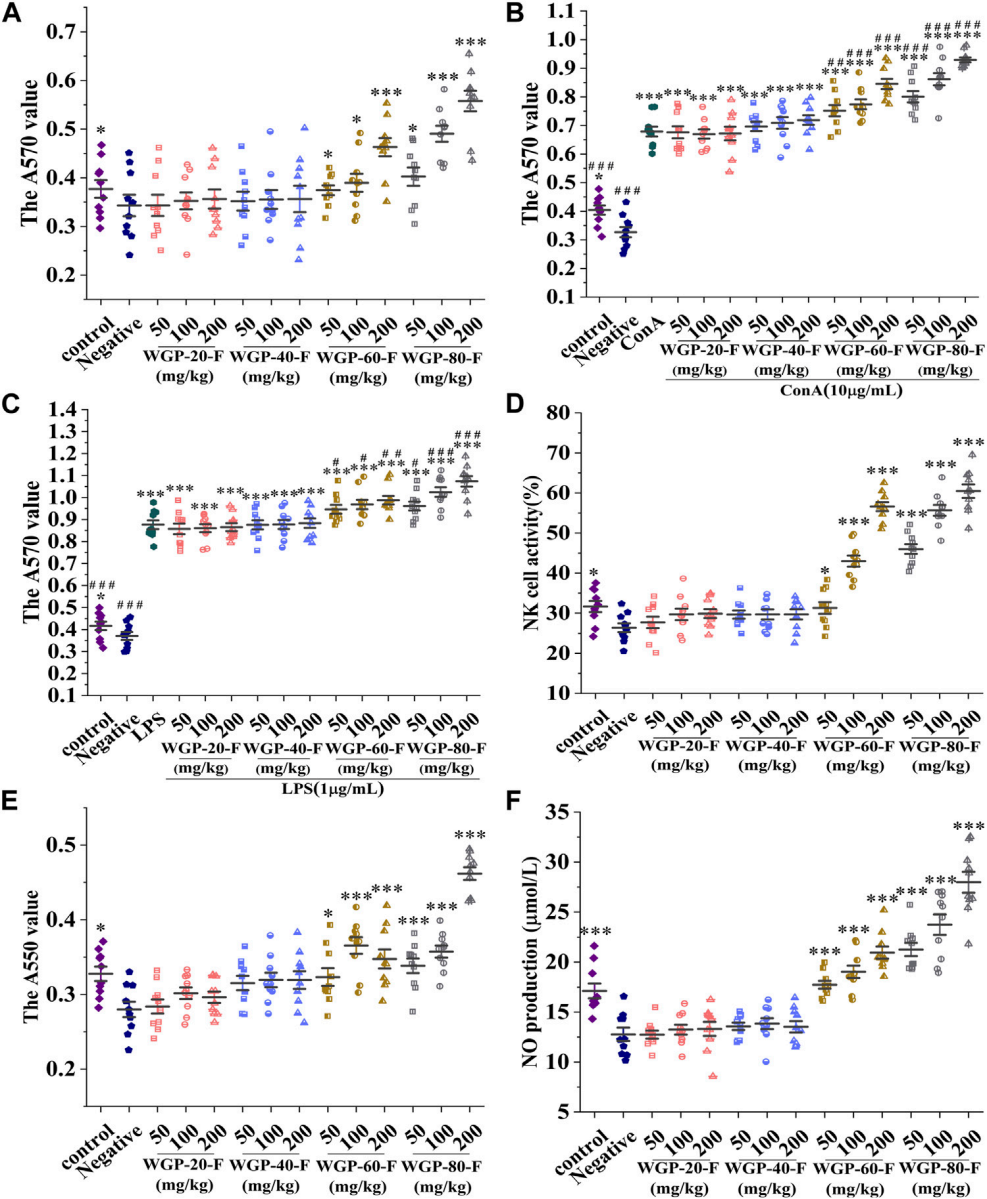
Modulatory effects of WGPs on immune cells. **(A)** Lymphocyte proliferation; **(B)** Synergistic Effect of WGPs and ConA on T Lymphocyte Proliferation; **(C)** Synergistic Effect of WGPs and LPS on B Lymphocyte Proliferation; **(D)** NK cell activity; **(E)** Phagocytic activity of macrophages; **(F)** NO produvtion. Every column represents the Mean ± SD. ***p < 0.001, **p < 0.01, *p < 0.05 *versus* negative group; ^###^ *p* < 0.001, ^##^ *p* < 0.01, ^#^ *p* < 0.05 *versus* ConA or LPS group.

As shown in Figures 7A–C, WGP-60-F and WGP-80-F had higher effects on lymphocyte proliferation than the negative control group within the dose range and could promote the proliferation of T lymphocytes and B lymphocytes induced by ConA or LPS in a dose-dependent manner (*p* < 0.05).WGP-80-F has the most significant effect on lymphocyte proliferation at the dose of 200 mg/kg; compared with that of the negative control group, the proliferation rate of WGP-80-F increased by 64.53% (*p* < 0.001); compared with those in the group treated with ConA or LPS alone, the proliferation rates of T lymphocytes and B lymphocytes in the WGP-80-F with ConA or LPS increased by 36.89% and 22.50%, respectively (*p* < 0.001).

#### 3.3.3 Effect of WGPs on the spleen NK cell activity

As shown in Figure 7D, compared with that in the negative control group, the NK cell activity in the WGP-60-F and WGP-80-F groups with dose-dependent supplementation increased significantly (*p* < 0.05). The activities of spleen NK cells in the WGP-60-F 200 mg/kg group and the WGP-80-F 200 mg/kg group could reach 56.60% and 60.47%, respectively. In comparison, the activities of spleen NK cells in the WGP-20-F and WGP-40-F groups had no significant change (*p* > 0.05).

#### 3.3.4 Effect of WGPs on the phagocytosis of peritoneal macrophages

Compared with those in the negative control group, the cells in the WGP-60-F and WGP-80-F groups indicated that the phagocytosis activity of macrophages enhanced significantly, as shown in Figure 7E (*p* < 0.05). The phagocytosis activity of WGP-80-F reached its highest values at the concentration of 200 mg/kg, which increased by 64.91% (*p* < 0.001). Compared with the negative control group, the WGP-20-F and WGP-40-F groups had no significant difference in the phagocytic capacity of macrophages (*p* > 0.05).

#### 3.3.5 Effect of WGPs on NO secretion

The NO secretion by WGPs with different fineness is shown in Figure 7F. The results showed that compared with the negative control group, WGP-60F and WGP-80-F remarkably enhanced the NO release of macrophages within the detected dose range (*p* < 0.001), among them, at a dose of WGP-80-F 200 mg/kg, the release of NO can reach 27.98 ± 3.33 μmol/L. However, the activity of NO in the WGP-20-F and WGP-40-F groups had no obvious activity (*p* > 0.05).

#### 3.3.6 Effect of WGPs on DTH in mice

**FIGURE 8:**
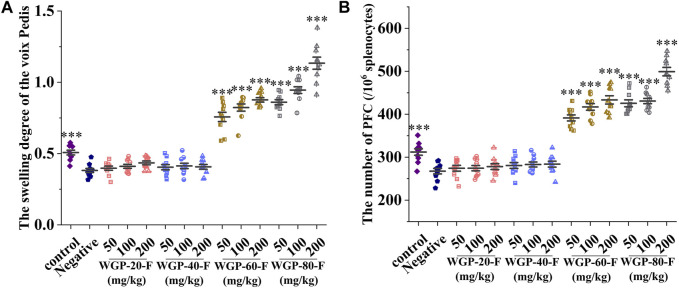
Modulating effects of ginseng on cellular and humoral immunity in mice. **(A)** Voix Pedis swelling; **(B)** The number of hemolytic plaque. Every column represents the Mean ± SD. ***p < 0.001, **p < 0.01, *p < 0.05 *versus* negative group.

As shown in Figure 8A, compared with that in the negative control group, the attacking part of the mice in WGP-60-F and WGP-80-F groups swelled obviously in a dose-dependent manner after the voix pedis in mice was stimulated with SRBC; the SRBC-immunised DTH reaction was markedly enhanced (*p* < 0.001). The swelling degree of WGP-80-F reached its highest value (1.13 ± 0.04 mm) at the concentration of 200 mg/kg. The voix pedis swelling degrees in the WGP-20-F and WGP-40-F groups were not obvious.

#### 3.3.7 Effect of WGPs on spleen PFC in mice

Compared with that in the control group, the number of PFC in the spleen cells in the negative control group mice after the SRBC stimulation significantly decreased, as shown in Figure 8B (*p* < 0.001). Compared with that in the negative control group, the number of haemolytic plaque generated by spleen cells in the WGP-60-F and WGP-80-F groups increased significantly (*p* < 0.001). The number of plaque in the WGP-80-F 200 mg/kg group mice increased significantly by up to 499 ± 31.05 per 10^6^ splenocytes.

## 4 Discussion

The gradient ethanol precipitation method is commonly used to classify polysaccharides by controlling ethanol concentration. In this study, four ginseng polysaccharides were obtained by adding 20%, 40%, 60%, and 80% ethanol for ethanol precipitation. Different fractions of ginseng polysaccharides derived via gradient ethanol precipitation have different primary structures. The *Mw* of each component of polysaccharides gradually decreases with the increase in alcohol precipitation concentration (Niu et al., 2023). WGP-20-F and WGP-40-F were glucans; t-ɑ-Glc*p* and (1→4)-ɑ-Glc*p* were their main sugar residues. When the ethanol precipitation concentration is 60%, small amounts of Ara, Gal and GalA were detected in WGP-60-F. Compared with WGP-60-F, WGP-80-F exhibited small amounts of Rha and GalA, with a Rha/GalA molar ratio of 1.0. Moreover, 40.66% of Ara was detected in WGP-80-Findicting that WGP-80-F contained the RG-I domain, which primarily subsisted with high-branched arabinan. The results showed that the increase in the ethanol concentration gradually decreased the *Mw* of polysaccharides and revealed a complex structure.

In a solution, polysaccharides may exhibit different conformations, such as helical, including single and triple helix, and random coils. The intramolecular and intermolecular hydrogen bonds are believed to be the major driving force for the formation of helix structures and triple chains of polysaccharides in water, respectively (Wang et al., 2008). The *Mw* of polysaccharides is also an important factor in the formation of triple-helix structures. In general, the glucan with *Mw* less than 5 × 10^4^ Da may not form a helix structure and can be easily formed with a *Mw* greater than 9 × 10^4^ Da (Wang and Zhang, 2009; Guo et al., 2021). In the present work, WGP-20-F and WGP-40-F with large *Mw* contain a triple-helix structure. However, WGP-60-F and WGP-80-F with small *Mw* do not have this structure. According to SEC-MALLS results, the U-shaped curve was observed in the <S^2^>_Z_^1/2^–*Mw* relationship curve of WGP-60-F and WGP-80-F, indicating they were highly branched polymers (Zhao et al., 2014; Zeng et al., 2016).

As a natural macromolecular active substance, polysaccharides can display immune enhancement by activating the immune-cell-dependent immune response system (Schepetkin et al., 2013; Lv et al., 2016). In the *in vivo* experiment, the body weight, spleen index and thymus index of mice in the WGP-60-F and WGP-80-F groups increased significantly at the dose of 200 mg/kg. Macrophages are important immune cells connecting nonspecific immunity and acquired immunity (Pan et al., 2013). WGP-60-F and WGP-80-F can significantly enhance the phagocytosis of macrophages at various doses and further promote the production of NO by regulating the immune activity of macrophages. The stimulating effect of polysaccharides on lymphocytes is mainly evaluated by inducing proliferation ConA is the mitogen of T lymphocytes. Moreover, LPS is a bacterial lipopolysaccharide, which is the mitogen of B lymphocytes. It can also stimulate the proliferation and differentiation of B lymphocytes. In this experiment, we also found that WGP-60-F and WGP-80-F can synergistically promote the proliferation of T/B lymphocytes with ConA and LPS in a dose-dependent manner. According to ICH guidelines, the enhancement of immune response may exaggerate hypersensitivity (EMA, 2005). In the WGP-60-F and WGP-80-F groups, the swelling degree of mice voix pedis and the number of haemolytic plaques were increased. This finding indicated that two polysaccharides could enhance the cellular and humoral immune functions by enhancing DTH reaction and PFC generation.

The primary structure of polysaccharides is an important factor affecting immune activity (Zhang et al., 2019). Low *Mw* polysaccharides have better water solubility and contain freer amino and hydroxyl groups within their molecules due to weak hydrogen bonding, which facilitates their active effects. However, high *Mw* polysaccharides are less likely to penetrate the cell membrane (Brasselet et al., 2019; Li et al., 2020; Zhu et al., 2021). Many scholars have also confirmed that Low *Mw* polysaccharides have a more significant effect in promoting lymphatic proliferation and activating macrophages (Yao et al., 2015b; Qian et al., 2021). In addition, monosaccharide composition and glycosidic bond are also an important factor affecting the immune enhancing activity of polysaccharides. Wang et al. found that in two types of Astragalus polysaccharides APS1 and APS2, two types, had a higher molecular weight and contained a higher AG domain. Although APS1 had a lower molecular weight, the proportion of AG domain was much lower than APS2. Immunological activity studies have confirmed that compared to APS1, APS2 exhibits stronger affinity for TLR4 and better immune activity. Therefore, it is speculated that the immune enhancing activity of Astragalus polysaccharides may be closely related to the AG domain (Wang et al., 2024).The polysaccharides from the *Polygala tenuifolia* Willd with the repeating unit of [→3)-α-Ara*f*-(1→ 3)-α-Ara*f*-(1→5)-α-Ara*f*-(1→5)-α-Ara*f*-(1→3)-α-Ara*f*-(1→]_n_ can activate the effector to release cytokines, the macrophages are activated to produce immune activity (Yu et al., 2023). This finding indicates that the structure of polysaccharides may have an impact on the immune activity of WGPs.

Different chain conformations of polysaccharides endow organisms with different functions and biological activities (Peng et al., 2005). In a solution, polysaccharides may exhibit different conformations, such as helical, including single and triple helix, and random coils, these structural characteristics can affect the direct contact between polysaccharides and immune cells, resulting in immune stimulating activity (Ferreira et al., 2015). In general, the triple-helix polysaccharide is considered to have multiple biological activities (Zhang et al., 2019; Meng et al., 2020; Hua et al., 2022). However, Li et al., 2020 found that two polysaccharides, MOP-2 and MOP-3, from *M. oleifera* leaves have similar monosaccharide composition and glycosidic bond types. However, MOP-2, which has no triple-helix structure, has good immunoregulatory activity. This may be due to the fact that the *Mw* and monosaccharide composition exceed the requirement of helical conformation for immunomodulatory activity. Immune stimulating polysaccharides with AG structure tend to exhibit random coil conformation (Ferreira et al., 2015). In this study, the *Mw* of WGP-20-F, WGP-40-F, WGP-60-F, and WGP-80-F decreased with increasing alcohol precipitation concentration, while the AG domain content was positively correlated with ethanol concentration. And among the four grades, WGP-80-F without triple-helix conformation also exhibited good immune enhancement activity. It can be inferred that lower *Mw*, higher AG content and complex glycosidic bonds may be key factors affecting the immune function of ginseng polysaccharides.

## 5 Conclusion

In summary, four polysaccharides, WGP-20-F, WGP-40-F, WGP-60-F and WGP-80-F, with different *Mw* of 1.720 × 10^6^, 1.434 × 10^6^, 4.225 × 10^4^, and 1.520 × 10^4^ Da, respectively were obtained from *P. ginseng* C. A. Meyer via gradient ethanol precipitation. WGP-20-F and WGP-40-F were glucans, and the four polysaccharides mainly comprised 4-ɑ-Glc*p*. WGP-60-F and WGP-80-F were acid heteropolysaccharides and also contain amounts of t-ɑ-Ara*f*, 5-ɑ-Ara*f* and 3,5-ɑ-Ara*f*, with complex glycosidic bonds. They were spherical branched structures without a triple-helix structure and also exhibited a good effect on immunoregulation. The research results provide a theoretical basis for the development of ginseng polysaccharides in health foods and the industrial production of ginseng active polysaccharides. However, the molecular mechanism of WGP-60-F and WGP-80-F participating in immune regulation still needs to research in further, and related research is ongoing.

## Data Availability

The original contributions presented in the study are included in the article/Supplementary Material, further inquiries can be directed to the corresponding author.
